# Association of electrolyte concentrations with pH of blood and urine of cows with left-sided abomasal displacement

**DOI:** 10.1093/jvimsj/aalag177

**Published:** 2026-08-24

**Authors:** Carina Oschlies, Barbara Riond, Christian Gerspach, Angelika Schoster

**Affiliations:** Food Animal Department, University of Zurich, Vetsuisse Faculty, 8057 Zurich, Switzerland; Food Animal Department, University of Zurich, Vetsuisse Faculty, 8057 Zurich, Switzerland; Food Animal Department, University of Zurich, Vetsuisse Faculty, 8057 Zurich, Switzerland; Equine Clinic, Ludwig-Maximilians-University, University of Munich, 85764 Oberschleissheim, Germany

**Keywords:** acid–base, paradoxic aciduria, Stewart-model, strong-ion difference

## Abstract

**Background:**

Ruminants with left-sided abomasal displacement (LDA) often develop metabolic alkalosis and paradoxical aciduria.

**Hypothesis/Objectives:**

To descriptively characterize electrolytes and acid–base variables in cows with LDA compared to healthy cows using the physicochemical (Stewart) approach, and to exploratively assess the influence of blood and urinary electrolytes on pH.

**Animals:**

Twenty healthy dairy cows and 20 cows with LDA.

**Methods:**

Retrospective cross-sectional study. Electrolytes, pH, total protein (TP), β-hydroxybutyrate (BHB), and glucose were measured in venous blood and urine. Base excess, anion gap, strong ion difference (SID), and strong ion gap (SIG) were calculated. Associations were evaluated using 2 stepwise forward multiple linear regression models.

**Results:**

Cows with LDA had significantly lower venous concentrations of sodium, chloride, potassium, magnesium, and calcium (*P* = .0013, *P* = .0023, *P* < .001, *P* < .001, *P* < .001), higher BHB (*P* < .001), and higher blood pH (*P* = .003) compared to controls. Calculated SID_3_, SID_4_, SID_5_, and SIG did not differ significantly between groups (*P* = .2, *P* = .37, *P* = .40, *P* = .62). In urine, cows with LDA showed significantly lower concentrations of Na^+^, K^+^, Cl^−^, Mg^2+^, and HCO₃^−^ (*P* = .0007, *P* < .001, *P* < .001, *P* = .004, *P* < .001). Urinary SID_3_ and SID_5_ were also significantly lower (both *P* < .001).

**Conclusions and clinical importance:**

Acid–base disturbances in cows with LDA can be interpreted using the physicochemical approach. Alterations in electrolyte concentrations contribute to changes in pH.

## Introduction

Left-sided abomasal displacement (LDA) is a common disorder in postpartum dairy cows. Since its first description in the 1950s^1^ LDA has been associated with characteristic electrolyte and acid–base abnormalities, collectively referred to as abomasal reflux syndrome.^2^ Reduced abomasal emptying rate leads to the reflux of abomasal contents—particularly chloride and hydrogen ions—into the rumen. This results in higher ruminal chloride concentrations and lower blood chloride concentrations due to impaired intestinal absorption.^3^^,^^4^ Cows with LDA often exhibit hypokalemia, hypochloremia, and metabolic alkalosis.^5^ Affected cows frequently develop aciduria, which in conjunction with metabolic alkalosis led to the description paradoxical aciduria.^6^ One proposed mechanism involves the renal excretion of hydrogen ions instead of potassium,^7^ although this has only been investigated in sheep.^8^^,^^9^ The pathophysiology of paradoxical aciduria in cattle remains poorly understood.

Acid–base status can be evaluated using 2 approaches: the traditional Henderson-Hasselbalch method and the physicochemical (Stewart) approach.^10–15^ The physicochemical approach offers the advantage of a mechanistic view as to why pH is changing as it fully integrates electrolyte and acid–base physiology.^16^ According to the physiochemical approach the plasma pH is determined by 3 independent factors: *p*CO_2_, net strong ion charge and the total plasma concentration of nonvolatile buffers (A_tot_, albumin, globulin, and phosphate). In plasma, strong ion difference (SID) is defined as the difference between fully dissociated strong cations and anions, mainly sodium, potassium, and chloride.^12^^,^^17^ Urine pH is influenced by 4 independent variables: SID, ammonium (NH_4_^+^), phosphate (PO_4_), and pCO_2_. In herbivores, high urinary potassium excretion makes potassium a major determinant of urine pH; high potassium concentration lead to alkaline urine and low potassium concentrations lead to acidic urine.^18^ The physiochemical approach has been applied to acid–base disorders in horses,^19^^,^^20^ foals,^21^ calves with diarrhea,^22^ and dogs.^15^ It has also been used to evaluate acidifying diets for preventing milk fever in dairy cows,^18^ but not to characterize acid–base alterations in cows with LDA. The primary aim of the study was to descriptively characterize electrolytes and acid–base variables in cows with LDA compared to healthy cows using the physicochemical (Stewart) approach. An additional exploratory aim was to investigate the influence of blood and urinary electrolytes on the respective pH values in these cows.

## Materials and methods

The study was approved by the veterinary authorities of the Kanton Zurich, Switzerland (approval number ZH 192/18).

### Animals

Twenty healthy cows (control group) and twenty cows with left-sided abomasal displacement (LDA) were included in the study. Control cows were selected from a teaching herd (Strickhof Lindau, Switzerland). Inclusion criteria for the control group were clinical examination without detectable abnormalities and age > 1 year. Cows in the LDA group were animals presented to the Ruminant Clinic, Department of Internal Medicine, University of Zurich, between 2018 and 2020. Inclusion criteria for this group were age > 1 year and a diagnosis of LDA confirmed by transabdominal ultrasonography.

### Samples

A venous blood sample was obtained from the animals in the control group via jugular venipuncture using an 18-gauge needle. For blood gas analysis, the sample was collected anaerobically into a spray-dried, calcium-balanced heparin syringe (BD A-Line, syringe for arterial blood gas analysis, DRIHEP A-Line, 3 mL, Becton Dickinson and Company, Plymouth, UK). From the same puncture site, an additional blood sample was drawn using a 10 mL syringe and transferred into a heparinized tube (Sarstedt AG, Nümbrecht, Germany). Venous blood samples from animals in the LDA group were collected via a venous catheter placed in the jugular vein. Urine samples were obtained from all cows via urinary catheterization. The vulva was cleansed with iodine soap and disinfected with an iodine solution before sampling. A flexible urinary catheter (Rüsch®, Germany) was inserted into the bladder. Urine was aspirated using a 60 mL syringe and immediately transferred into a heparin syringe (BD A-Line, syringe for arterial blood gas analysis, DRIHEP A-Line, 3 mL, Becton Dickinson and Company, Plymouth, UK), then sealed airtight with a cap. All samples from the control group were placed on ice, transported to the laboratory, and analyzed within 3 h.

Urine samples from the LDA group were analyzed immediately.

### Methods

The venous blood gas samples from both groups were analyzed with the RAPIDPoint 500e (Siemens Healthineers AG, Zurich, Switzerland) which measures pH, *p*CO_2_, *p*O_2_, sodium, potassium, chloride, ionized calcium, L-lactate, and, derives HCO_3act_, HCO_3std_, and calculates anion gap and base excess (BE). A standardized analysis temperature of 37 °C was used. Beta-hydroxybutyrate (BHB) concentrations were determined using a handheld beta-ketone testing device (BHB-Check, Pharmadoc GmbH, Germany). Magnesium, phosphorus, and total protein concentrations were measured from heparinized plasma using the cobas® 6000 analyzer series, cobas® c 501 module (Roche Diagnostics, Basel, Switzerland).

The following variables were assessed semi-quantitatively in urine using Combur Test strips (Roche Diagnostics): pH, acetoacetate, and glucose. Urine specific gravity was measured with a manual handheld refractometer. The concentrations of HCO_3_^−^, sodium, potassium, chloride, magnesium, phosphorus, calcium, and total protein were determined using the cobas® 6000 analyzer series, cobas® c 501 module.

For the evaluation of the acid–base status in blood plasma the following variables were calculated: BE (automatically calculated by the RAPIDPoint 500e) using BE_ecf_ = HCO_3act_ − 24.8 + 16.2× pH (37) − 7.40 (reference value 2 − 8 mmol/L)^23^; Anion gap (AG) = (Na^+^ + K^+^) − (Cl^−^ + HCO_3_^−^; reference value 15-20 mmol/L^24^); SID_3_ = (Na^+^ + K^+^) − Cl^−^ (reference value 41-45 mEq/L); SID_4_ = (Na^+^ + K^+^) − (Cl^−^ − lactate^−^; reference value 39-43 mEq/L)^25^ and SID_5_ = (Na^+^ + K^+^ + Ca^+^) − (Cl^−^ − lactate; reference value 40-50 mEq/L)^26^; Total concentration of nonvolatile weak buffers A_tot_ = 0.343× TP (g/L).^22^ The anion charge of non-volatile weak acids (A^−^) [A_tot_/(1 + 10^(7.08−pH)^]^25^; Strong ion gap (SIG) = AG − A^−^^26^ (reference value +2 to −6 mEq/L).^23^ Ionized calcium concentration was adjusted for pH in bovine plasma using the experimentally determined equation _i_Ca^2^  _7.4_ = _i_Ca^2+^  _measured_ ×10 ^(−0.23 × (7.4−pH measured))^.^27^ For the evaluation of urine composition, the following SIDs were calculated: SID_3urine_ = (Na^+^ + K^+^) − Cl^−^^28^; For SID_5urine_, a simplified version of the formula used by Constable et al. was applied: SID_5urine_ = (Na^+^ + K^+^ + Mg^+^ + Ca^+^) − Cl^−^.^18^

### Statistical analysis

Data are presented as mean and standard deviation. Not normally distributed data are presented as median and range. Normality of the data was tested using the Shapiro–Wilk test and a visual graphical inspection using Q–Q-plots. Differences between groups were assessed using an unpaired *t-*test and for not normally distributed data the Mann–Whitney *U* test. Differences were classified as significant if *P* < .05. No formal adjustment for multiple testing was applied. These statistical analyses were performed using the commercial statistical software program GraphPad Prism.

Two multivariable linear regression models with stepwise forward selection (IBM SPSS Statistics 29.0.1.0) were used to assess associations between acid–base variables and electrolytes. Dependent variables were venous blood pH (Model 1) and urinary pH (Model 2). Candidate predictors included blood (Na, K, Cl, Mg, P, Ca, BHB), and urine electrolytes (Na, K, Cl, Mg, P, Ca), and study group (0 = LDA, 1 = control).

Multicollinearity was assessed using a correlation matrix; for pairs with Pearson *r* > 0.70, one variable was retained. Final predictors were blood Na, Cl, Mg, Ca, BHB; urine Na, K, Mg, P, Ca; and group. Stepwise criteria were *P* ≤ .05 (entry) and *P* ≥ .10 (removal). Model performance was evaluated by *R, R*^2^, adjusted *R*^2^, partial *R*^2^, and VIF.

Assumptions were assessed via residual diagnostics (normality: Shapiro–Wilk, P–P plots, histograms; homoscedasticity: residuals vs. predicted; influence: standardized/studentized residuals, Cook’s distance). Both models met assumptions. For blood pH: residuals within acceptable ranges (standardized –1.864 to 1.909; studentized −2.101 to 2.336), normality supported (*P* = .703), no heteroscedasticity, low Cook’s distance (max 0.302). For urine pH: residuals acceptable (standardized −2.002 to 1.971; studentized −2.401 to 2.167), normality supported (*P* = .790), no heteroscedasticity, and generally low Cook’s distance (mean 0.066), with one higher value (1.581) without apparent influence.

## Results

The cows in the control group had a mean age of 4.6 ± 1.8 years, while cows with LDA had a mean age of 4.6 ± 1.7 years. Multiple breeds were represented in both groups. In the control group, 8/20 (40%) were Brown Swiss, 8/20 (40%) Holstein Friesian, and 4/20 (20%) Red Holstein. In the LDA group, 5 different breeds were included: Holstein Friesian 9/20(45%), Red Holstein 6/20 (30%), Simmental 3/20 (15%), 1 Brown Swiss cow (5%), and 1 mixed-breed cow (5%; Supplementary Table 1). Cows in the LDA group were, on average, 12.7 days in milk (DIM; SD = 5.3), whereas cows in the control group were 204.4 DIM (SD = 128.6). In the LDA group, 11 cows had been treated for ketosis by administration of dexamethasone, glucose (IV), or propylene glycol (PO); however, only 2 cows received dexamethasone and 4 cows received glucose infusion on the day of referral. The remaining treatments were administered at least 2 days before referral. None of the cows had received an acidogenic diet for hypocalcemia prevention before calving, nor had any received glucocorticoids with clinically relevant mineralocorticoid effects.

**Figure 1:**
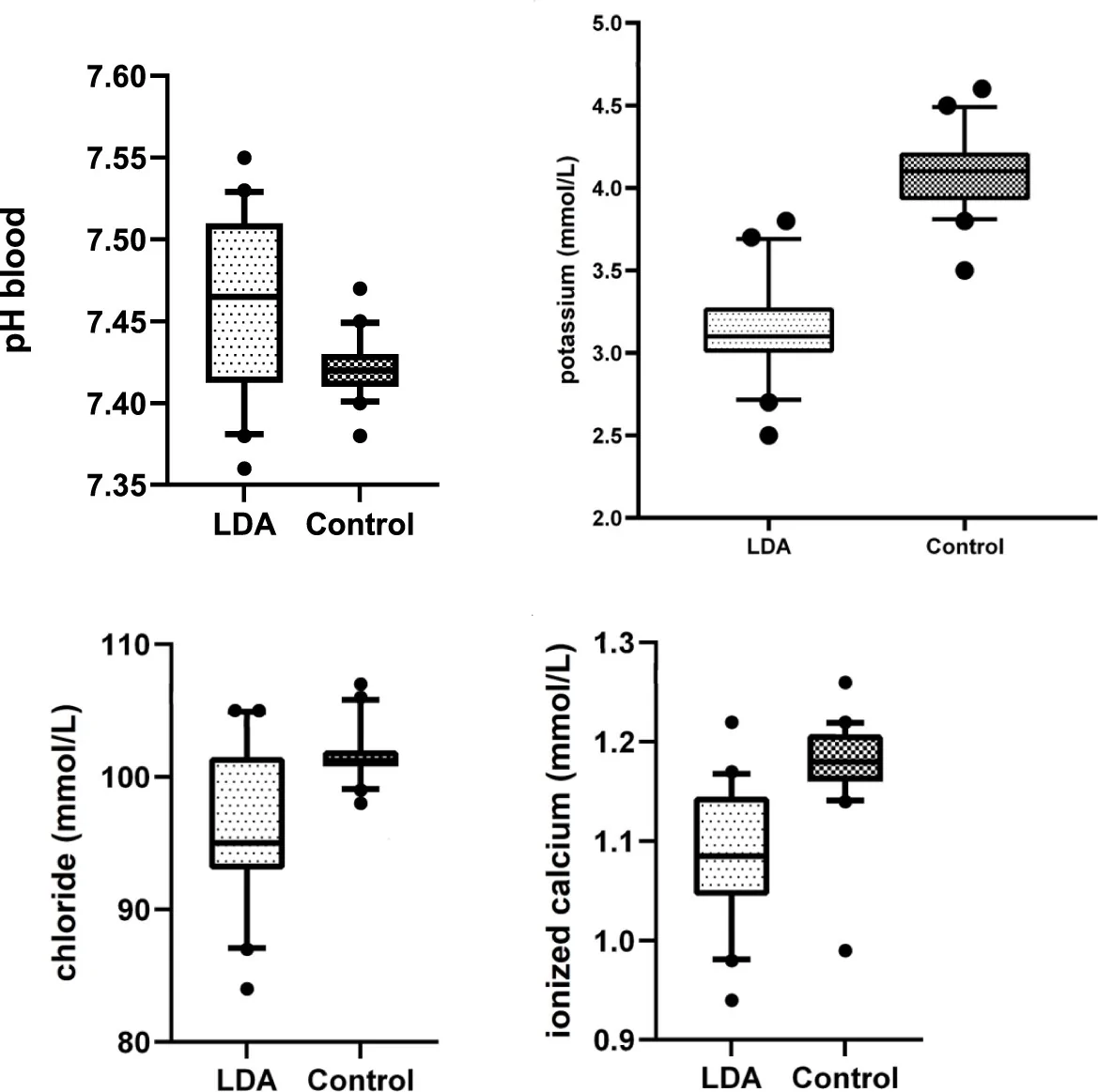
Selected venous blood variables of healthy cows (*n* = 20) and cows with LDA (*n* = 20). The boxes represent the 25th to 75th percentile, the whiskers represent the 10th to 90th percentile, outliers are presented as dots. Abbreviation: LDA, Left abomasal displacement.

The results of the plasma acid–base assessment are presented in Table 1. There were no cases of strong ion acidosis in the cows with LDA. In contrast, 8 cows in this group had a strong ion alkalosis, and 1 cow had an alkaline blood pH with pCO_2_, BE, AG, and SIDs within their respective reference intervals. Two cows in the LDA group had an higher anion gap, while the remaining 18 animals had an anion gap within the reference range. Seven animals had a BE, 8 had a base deficit, and in the remaining cows the BE was within the reference interval. All cows in the LDA group exhibited hypokalemia (reference range 3.9-5.0 mmol/L). 6/20 (30%) of the cows with LDA had hypochloremia (reference range: 94-102 mmol/L), and 9/20 (45%) had an alkalotic venous blood pH(reference range: 7.35-7.45) accompanied by elevated bicarbonate concentrations (reference range: 22-26 mmol/L). Potassium and chloride concentrations were significantly lower in the LDA group compared to controls (*P* < .001 and *P* = .0023). Blood pH was significantly higher in LDA cows compared to controls (*P* = .003; 95% CI, −0.07 to −0.01; Figure 1). Sodium, magnesium, and ionized calcium concentrations were also significantly lower in the LDA group (*P* = .0013, *P* < .001, and *P* < .001, respectively). No significant difference in bicarbonate concentrations was observed between groups (*P* = .14). None of the 3 calculated SIDs was significantly different between groups.

**Table 1:** Venous blood variables of healthy cows (*n* = 20) and cows with left sided abomasal displacement (*n* = 20).

Variable (unit)	LDA	Control	*P-value*	95% CI
**Na (mmol/L)**	137.1 (2.7)	139.7 (2.0)	.0013	1.1-4.1
**K (mmol/L)**	3.2 (0.4)	4.1 (0.2)	<.001	0.8-1.2
**Cl (mmol/L)** ^**a**^	95 (84-105)	101 (98-107)	.0023	2.0-8.0
**Mg (mmol/L)**	0.73 (0.14)	1.05 (0.07)	<.001	0.25-0.34
**Ph (mmol/L)**	1.2 (0.56)	1.45 (0.28)	0.08	−0.03 to 0.53
**Ionized Ca (mmol/L)** ^**a**^	1.09 (0.94-1.22)	1.20 (1.00-1.30)	<.001	0.06-0.13
**Adjusted ionized Ca (mmol/L)** ^**a**^	1.12 (1.0-1.27)	1.12 (1.0-1.29)	<.001	0.04-0.11
**TP (g/L)** ^**a**^	76.0 (55.0-92.0)	76.5 (69.0-101.0)	0.78	−5.0 to 8.0
**HK (%)**	34 (5.7)	–	–	–
**BUN mmol/L)**	5.2 (2.3)	–	–	–
**Creatinine (μmol/L)**	85 (17)	–	–	–
**BHB (mmol/L)**	2.3 (1.1)	0.7 (0.1)	<.001	−2.1 to −1.1
**Glucose (mmol/L)** ^**a**^	5.4 (2.8-13.0)	3.4 (3.0-3.7)	<.001	−2.5 to −0.40
**pH**	7.46 (.06)	7.42 (.02)	.003	−0.07 to −0.01
***p*CO**_**2**_ **(mmHg)**	40.0 (5.8)	42.9 (4.7)	.01	−10.2 to −3.9
***p*O**_**2**_ **(mmHg)**	38.0 (4.5)	30.9 (5.2)	<.001	−10.2 to −3.9
**HCO**_**3**_ ^**−**^_**std**_ **(mmol/L)**	28.0 (5.5)	26.0 (1.9)	.14	−4.6 to 0.7
**BE**_**ecf**_ **(mmol/L)**	4.3 (6.1)	2.5 (2.2)	.22	−4.7 to 1.1
**AG (mmol/L)**	21.1 (3.7)	19.2 (2.2)	.06	−3.8 to 0.1
**L-Lactate (mmol/L)** ^**a**^	1.3 (0.3-3.8)	0.7 (0.2-1.9)	.0013	−0.9 to −0.2
**SID**_**3**_ **(mEq/L)**	44.1 (6.3)	42.1 (2.4)	.2	−5.1 to 1.1
**SID**_**4**_ **(mEq/L)**	42.7 (6.0)	41.4 (2.4)	.37	−4.2 to 1.6
**SID**_**5**_ **(mEq/L)**	43.8 (6.0)	42.6 (2.4)	.40	−4.1 to 1.7
**A**_**tot**_ ^**a**^ **(mmol/L)**	26.1 (18.9-31.2)	26.2 (23.7–34.6)	.78	−1.7 to 2.7
**A** ^**−**a^ **(mmol/L)**	18.6 (12.4-23.2)	18.1 (16.2–24.7)	.66	−1.5 to 1.1
**SIG** ^a^ **(mEq/L)**	−2.7 (−7.4 to 6.4)	−3.4 (−10.5 to −0.7)	.62	−2.5 to 1.7

Data presented as mean (standard deviation), 95% CI = 95% confidence interval of the difference.

SID_3_ = (Na^+^ + K^+^) − Cl^−^

SID_4_ = (Na^+^ + K^+^) − (Cl^−^ − lactate^−^)

SID_5_ = (Na^+^ + K^+^ + Ca^2+^) − (Cl^−^ − lactate^−^)

^a^Not normally distributed data as median (range).

**Figure 2:**
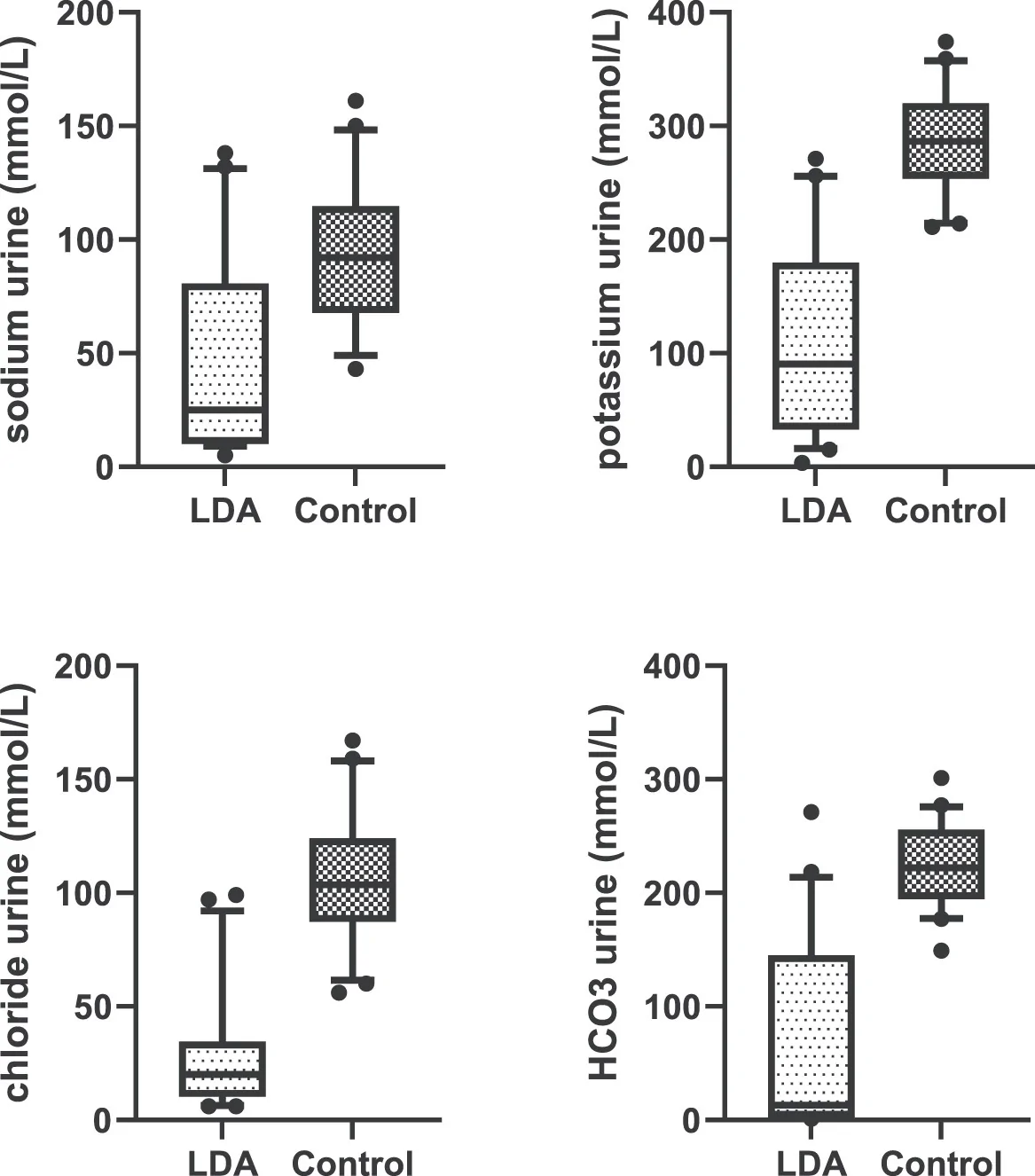
Selected urine acid base relevant variables of healthy cows (*n* = 20) and cows with left abomasal displacement (*n* = 20). The boxes represent the 25th to 75th percentile, the whiskers represent the 10th to 90th percentile, outliers are presented as dots. Abbreviations: LDA, Left abomasal displacement, HCO_3_, Bicarbonate.

The results of the acid–base analysis of urine are presented in Table 2. Cows with LDA had significantly lower concentrations of sodium, potassium, chloride, and magnesium in the urine (*P* = .0007, *P* < .001, *P* < .001, and *P* = .004, respectively) (Figure 2). No statistically significant difference in urine pH was observed between the groups. However, acidic urine (pH < 6.5; reference range: 7.0-8.7^29^) was observed only in the LDA group: 5/20 (25%) of the cows had a urinary pH of 5 and 2/20 (10%) had a pH of 6. In the control group, all cows had a urine pH of 7 or 8. Bicarbonate concentrations in urine were significantly lower in the LDA group (*P* < .001) (Figure 2). Both calculated SID_3urine_ and SID_5urine_ were significantly lower in the LDA group (both *P* < .001).

**Table 2:** Urine acid base variables of healthy cows (*n* = 20) and cows with left sided abomasal displacement (*n* = 20).

Variable (unit)	LDA	Control	*P*-value	95% CI
**Na (mmol/L)** ^**a**^	25.0 (5.0-138.0)	92 (43.0-161.0)	.0007	31.0-78.0
**K (mmol/L)** ^**a**^	101.0 (15.0-271.0)	286.5 (211.0-374.0)	<.0001	124.0-225.0
**Cl (mmol/L)** ^**a**^	20.0 (6.0-99.0)	104 (56.0-167.0)	<.0001	66.0-98.0
**Mg (mmol/L)** ^**a**^	3.41 (0.56-31.9)	10.51 (3.28-17.32)	.004	2.26-8.34
**Ph (mmol/L)** ^**a**^	1.1 (1.1-7.8)	1.1 (1.1-1.1)	.11	0.0-0.0
**Total Ca (mmol/L)** ^**a**^	0.4 (0.2-19.7)	0.3 (0.2-1.6)	.69	−1.9 to 0.06
**TP (g/L)** ^**a**^	0.46 (0.17-1.38)	0.45 (0.28-0.77)	.58	−0.15 to 0.06
**Acetoacetate (mg/dL)** ^**a**^	15 (0.0-800)	0.0 (0.0-0.0)	<.0001	−400 to −15.0
**Glucose (mg/dL)** ^**a**^	0 (0.0-300)	0 (0.0-0.0)	.23	0.0-0.0
**pH** ^**a**^	7.5 (5.0-9.0)	8.0 (7.0-8.0)	.52	−1.0 to 2.0
**HCO**_**3**_^**−**^ **(mmol/L)**^**a**^	13.2 (1.2-271.2)	222.0 (149.0-301.0)	<.0001	128.0-206.0
**SID**_**3**_ **(mEq/L)**	138.5 (107.6)	273.3 (47.9)	<.0001	81.4-188.1
**SID**_**5**_ **(mEq/L)**	148.3 (107.3)	284.0 (48.7)	<.0001	82.4-189.1
**USG**	1024 (14)	–	–	–

Data presented as mean (standard deviation), 95% CI = 95% confidence interval of the difference.

SID_3_ = (Na^+^ + K^+^) − Cl^−^

SID_5_ = (Na^+^ + K^+^ + Mg^+^ + total Ca) − Cl^−^

^a^Not normally distributed data as median (range).

The results of the regression analysis are presented in Tables 3 and 4.

**Table 3:** Multiple linear regression coefficients for predictors of venous blood pH in healthy cows and cows with LDA (*n* = 40).

Predictor	Beta	*P*-value	*R* ^2^	T	VIF
**Group**	−.969	<.001	0.435	0.44	2.26
**Blood Sodium**	.376	.008	0.178	0.83	1.21
**Blood BHB**	−.491	.010	0.171	0.46	2.16

Final Model with venous blood pH as dependent variable; *R* = .679; *R*^2^ = .460 with a 95% CI of 0.18-0.67; adjusted *R* = .416; *F* (3.36) = 10.24; *P*-value < .001.

**Table 4:** Multiple linear regression coefficients for predictors urine pH in healthy cows and cows with LDA (*n* = 40).

Predictor	Beta	*P*-value	R ^2^	T	VIF
**Urine potassium**	.917	<.001	0.457	0.35	2.86
**Group**	−.967	<.001	0.408	0.26	3.88
**Urine Sodium**	.383	.004	0.217	0.66	1.51
**Blood BHB**	−.404	.010	0.176	0.46	2.18

Final Model with urine pH as dependent variable; *R* = .806; *R*^2^ = .65 with a 95% CI of 0.44-0.80; adjusted *R* = .61; *F* (4.35) = 16.25; *P*-value < .01.

**Figure 3:**
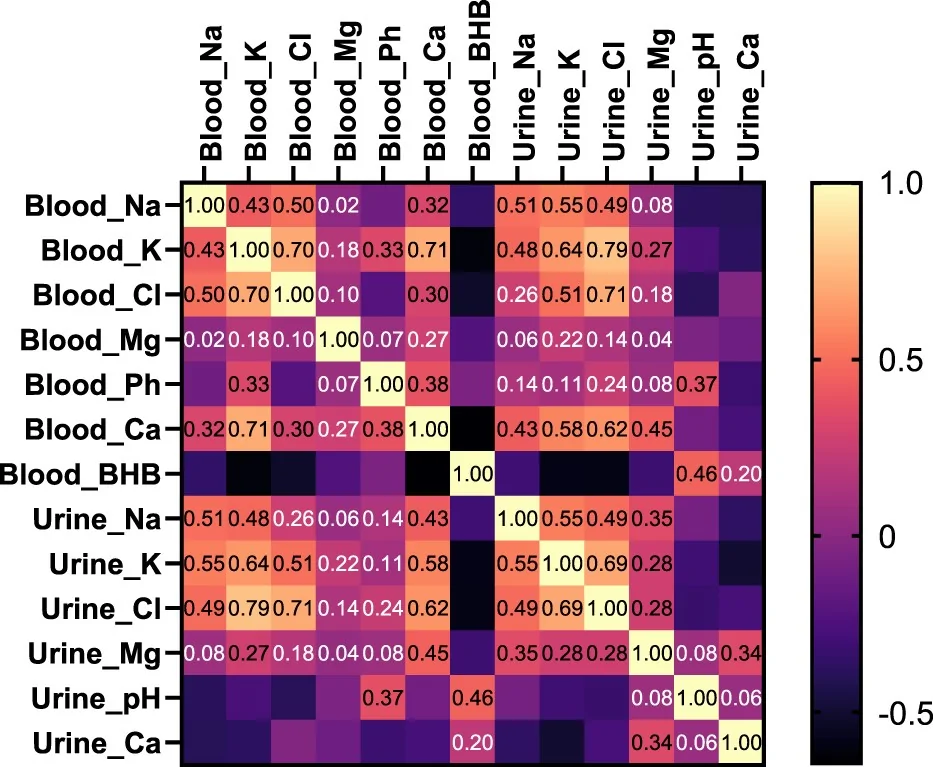
Heatmap of correlation coefficients among measured variables.

In Model 1 (full model presented in Table 5) a forward stepwise multivariate linear regression was performed with venous blood pH as dependent variable. The results of the correlation analysis are presented as a heatmap in Figure 3. After exclusion of collinear predictors (*r* > .7), the final model included Group, blood sodium, and blood BHB concentrations. The model significantly predicts venous blood pH with F (3.36) = 10.24 (*P* < .001) and explained 46% of the variance in venous blood pH (R^2^ = 0.46 with a 95% CI, 0.18-0.67; adjusted *R*^2^ = .416, *P* < .001) VIF were all < 2.2, and partial *R*^2^ values indicated the relative contribution of each variable. All predictors were significant: Belonging to the LDA group was associated with a significantly higher blood pH (β = −0.969, *P* < .001)*.* Higher blood sodium concentrations were associated with higher venous blood pH (β = 0.376, *P* = .008) and a higher venous blood BHB was associated with a lower blood pH (β = −0.491, *P* = .01). No issues of multicollinearity were observed.

**Table 5:** Full multivariable linear regression model for venous blood pH in healthy cows and cows with left displaced abomasum (LDA) (*n* = 40).

Predictor	B	SE	β	*t*	*P*-value
**Intercept**	7.043	0.369		19.069	<.001
**Blood Na**	0.006	0.002	0.370	2.753	.010
**Blood K**	−0.025	0.022	−0.312	−1.138	.264
**Blood Cl**	−0.002	0.002	−0.181	−.928	.360
**Blood Mg**	−0.002	0.004	−0.078	−.660	.514
**Blood Ph**	−0.026	0.016	−0.261	−1.613	.117
**Blood Ca**	−0.117	0.090	−0.192	−1.298	.204
**Blood BHB**	−0.017	0.007	−0.410	−2.378	.024
**Group**	−0.033	0.026	−0.361	−1.259	.217
**Residual diagnostics summary**					
**Metric**	**Min**	**Max**		**Mean**	**SD**
**Residual**	−0.0606	0.0621		0.0000	0.0290
**Standardized residual**	−1.864	1.909		0.000	0.892
**Studentized residual**	−2.101	2.336		−0.002	1.034
**Cook’s distance**	0.000	0.302		0.044	0.066
**Leverage**	0.038	0.971		0.200	0.167

In Model 2 (full model presented in Table 6) a forward stepwise multivariate linear regression was performed with urinary pH as dependent variable. The results of the correlation analysis are presented as a heatmap in Figure 3. After exclusion of collinear predictors (*r* > .7), the final model included urinary potassium, Group, urinary sodium, and venous blood BHB concentration. The model significantly predicts urinary pH with F(4.35) = 16.248 (*P* < .001) and explained 61% of the variance in urinary pH (*R*^2^ = 0.65 with a 95% CI, 0.44-080; adjusted *R*^2^ = 0.610, *P* < .001). VIF were all <3.8 and partial *R*^2^ values indicated relative contribution of each variable. All predictors were significant: Higher urine potassium concentrations were associated with higher urinary pH (β = 0.917, *P* < .01). Belonging to the LDA group was associated with significantly lower urinary pH (β = −0.967, *P* < .001). Higher sodium concentrations in the urine were associated with a higher urinary pH (β = 0.383, *P* = .004) and higher venous BHB was associated with a lower urinary pH (β = −0.404, *P* = .010). No issues of multicollinearity were observed.

**Table 6:** Full multivariable linear regression model for urinary pH in healthy cows and cows with left displaced abomasum (LDA) (*n* = 40).

Predictor	B	SE	β	*t*	*P*-value
**Intercept**	8.752	0.709		12.338	<.001
**Blood BHB**	−0.312	0.163	−.282	−1.916	.065
**Group**	−1.333	0.507	−.552	−2.628	.013
**Urine Na**	0.009	0.003	.358	3.007	.005
**Urine K**	0.008	0.002	.781	4.587	<.001
**Urine Cl**	−0.012	0.004	−.486	−3.094	.004
**Urine Mg**	0.024	0.025	.129	0.971	.339
**Urine pH**	−0.205	0.105	−.214	−1.947	.061
**Urine Ca**	−0.082	0.043	−.259	−1.891	.068
**Residual diagnostics summary**					
**Metric**		**Min**	**Max**	**Mean**	**SD**
**Residual**		−1.3470	1.3134	0.0000	0.5940
**Standardized residual**		−2.022	1.971	0.000	0.892
**Studentized residual**		−2.401	2.167	0.013	1.033
**Cook’s distance**		0.000	1.581	0.066	0.250
**Leverage**		0.037	0.727	0.200	0.162

## Discussion

In the present study, cows with LDA had a significantly higher venous blood pH and significantly lower plasma potassium and chloride concentrations compared to the control group. Due to the reflux of abomasal contents into the rumen, chloride ions are no longer available for exchange with bicarbonate in the small intestine, leading to a decrease in blood chloride concentration. This abomasal reflux syndrome has been confirmed in other studies as well, although the severity of the findings varies.^30^^,^^31^ In the present study, 30% had hypochloremia. In another study 43% of the cows had lower plasma chloride concentrations.^30^ In that study a cutoff of 95 mmol/L was used for hypochloremia. In the present study, 3 cows had a serum chloride concentration of 94 mmol/L. Therefore, the findings of the 2 studies are comparable.

Regarding blood potassium concentrations similar results were reported in a retrospective study of 1982 cows with displaced abomasum. In that study, 82% of the cows with LDA had hypokalemia (plasma potassium concentration < 4 mmol/L).^30^ In the present study, all cows with LDA (100%) were affected. In another study, 55% of cows with LDA had hypokalemia, even though nearly the same reference cutoff for potassium (<3.9 mmol/L) was used.^32^

As an explanation for the hypokalemia, there are reduced feed intake and shift of potassium into the cells due to alkalosis.^33^^,^^34^ As 6 cows received treatment with glucose, dexamethasone, or both on the day of referral, hyperglycemia might represent an additional contributing factor to the observed hypokalemia.^32^ Notably, cows in the LDA group exhibited significantly higher blood glucose concentrations compared to control cows (*P <* .001).

The venous blood pH of the cows in the LDA group, with a mean of 7.46, was comparable to another large study. In that study, cows with left-sided abomasal displacement had a median blood pH of 7.43; 36% of the cows had a blood pH greater than 7.45.^30^

The blood sodium concentration was significantly lower in the LDA group than in the healthy group (*P =* .0013). It is important to emphasize that the lower concentration of sodium in the LDA group is per definition not a hyponatremia (sodium < 132 mmol/L). There has also been a significant higher L-concentration in the LDA group (1.3 (0.3-3.8) vs 0.7 (0.2-1.9) mmol/L, *P =* .001). A higher L-lactate concentration occurs in cows with abomasal volvulus due to hypoperfusion caused by elevated intraluminal pressure. In cows with left displaced abomasum, however, the rise in intraluminal pressure is not sufficient to account for the observed elevation in L-lactate.^35^ In another study, a higher blood L-lactate concentration in cows with LDA was also reported. In that study, 23% of LDA cases had an plasma L-lactate value greater than 2.2 mmol/L, the L-lactate concentration in cows with abomasal volvulus was markedly higher than in cows with LDA (median 5.1 vs. 1.3 mmol/L), and 42.6% of the cows with abomasal volvulus had hyper-L-lactatemia.^30^ The higher blood L-lactate concentration in cows with LDA had only a minor effect on the AG or the SIG. The anion gap was marginally higher in the LDA group (21.1, SD = 3.7) compared with the control group (19.2, SD = 2.2). Only 2 cows in the LDA group showed an AG above the reference range (21.7 and 27.4 mmol/L). These 2 cows also had elevated L-lactate, higher plasma BHB concentrations or both, which could explain the higher AG. However, when considering the SIG, cows in the control group had slightly lower SIG values than cows in the LDA group. In the control group, all 20 animals had L-lactate and BHB concentrations within the reference intervals (<2 and <1 mmol/L, respectively). Therefore, it appears that the control group had a higher concentration of unidentified strong anions in the blood. With a clinically relevant increase in L-lactate, the AG would be expected to increase and the SIG to decrease. This effect on AG and SIG was demonstrated in another study, in which calves with diarrhea were retrospectively evaluated. In this study, the SIG and AG were strongly associated with the acid–base status and the severity of the calves’ clinical presentation, although in these calves not only L-lactate but also D-lactate plays a role.^36^

Cows in the LDA group had significantly lower ionized calcium concentrations in blood compared to the control group (*P* < .001). However, only 4 cows in the LDA group were slightly hypocalcemic (blood ionized calcium 0.8-1.05 mmol/L) based on the reference interval reported in one study. All other cows had calcium values above 1.05 mmol/L and were therefore within the reference range.^37^ In our study, all cows with mild hypocalcemia were also hypokalemic, suggesting that hypokalemia might have contributed to the observed decrease in ionized calcium, as reported in the study by Karapinar. In this study total calcium and plasma chloride concentration showed the strongest association with ionized calcium in adult cattle with various clinical disorders. Plasma potassium concentration was also positively associated with ionized calcium in sick adult cattle, explaining approximately 9% of the variation in ionized calcium concentrations in the mentioned study.^38^ The lower plasma phosphorus concentrations (*P* = .083) observed in the LDA group could be attributable to reduced feed intake.^39^ Two cows received dexamethasone and 4 cows received glucose infusion on the day of referral. Intravenous treatment with a dextrose solution might have influenced blood concentrations of BHB and phosphorus. This type of treatment decreases BHB concentrations for approximately 12 h and increases blood glucose levels for about 2 h. Since more than 2 h passed between admission of the animals to the clinic and blood sampling, the treatment in the present study likely only affected BHB concentrations. Accordingly, BHB concentrations were markedly lower in these 4 cows. Phosphorus concentrations could also be lower for up to 12 h after dextrose treatment. Therefore, phosphorus levels in these 4 cows might also have been lower as a result.^40^ The BE was higher in the LDA group, although not significantly. In combination with a higher pH, this suggests the presence of a metabolic alkalosis. This is consistent with another study in which cows with LDA all had BE values within the reference range. That study also showed that cows with abomasal volvulus had significantly higher BE values.^35^

Although 50% of the cows in the LDA group had a SID_3_ > 45 mEq/L and 60% had a SID_4_ > 43 mEq/L, the authors suggest that the electrolyte changes were not pronounced enough to reach statistical significance. Since chloride ions are the main determinant of SID in LDA, and hypochloremia in the present study was not pronounced, some cows also had SID values within the reference range.

Briefly, SID indicated that some cows in the LDA group exhibited a strong ion alkalosis, while SIG and AG suggested the presence of elevated unmeasured anions in others. Overall, the acid–base disturbances observed in this study were mild.

The cows with LDA had a significantly lower sodium, chloride, and potassium concentration in the urine (*P =* .0007, *P* < .001, and *P* < .001).

The lower urinary sodium concentration in the LDA group (25.0 (5.0-138.0) mmol/L) was because sodium is reabsorbed together with water in the kidneys to maintain blood pressure during dehydration.

Two independent factors influence electrolyte transport in the kidneys of rats: urinary electrolyte concentration and hydration state.^41^ The lower potassium and chloride concentrations are possibly due to lower blood concentrations of these electrolytes. Electrolyte excretion also depends on the diet and the time of sampling after feeding. The greatest urinary sodium concentration variation in dogs occur in low and very low sodium diets and when samples were taken directly after eating.^42^

The significantly lower SID_3_ and SID_5_ in the urine of cows with LDA (*P* < .001) indicates that one of these ions must play a major role in affecting the urinary pH. Exploratory forward stepwise multivariable regression analysis suggested a positive association between urinary potassium concentration and urinary pH. This finding was also reported in another study. Potassium had the greatest effect on urinary pH, with a low potassium concentration leading to an acidic urine.^18^ Other studies also found, that the greatest influence on urinary pH variability has the potassium concentration in the urine.^18^^,^^43^ In another study, the fractional urinary excretion of potassium was evaluated in cows with LDA before and after surgical treatment. The fractional excretion of potassium was lower in cows with LDA than in the same cows after surgical correction of the LDA.^43^

The urine electroneutrality can be described with the following simplified equation: SID + (NH_4_^+^) − (HCO_3_^−^) = 0. For cattle ingesting a typical diet the urine is alkaline and the urine NH_4_^+^ is <7 mEq/L. Under these circumstances urinary SID approximates the urinary HCO_3_^−^.^18^ We were able to verify this in the present study for the animals in the control group: urinary HCO_3_^−^ was 222.0 (SD 152), the urinary SID_3_ 273.3 (SD 47.9), and the urinary SID_5_ 284 (48.7). When aciduria is present, NH_4_^+^ approximates not 0. In contrast there is a marked variability of ammonium in acidic urine. In the present study ammonium was not measured. As NH_4_^+^ is one of the 4 independent variables that influence the pH of the urine, this could have contributed to a lower urine pH in the LDA group.

The calculation of the SID of bovine urine with the formula (Na^+^ + K^+^ + Ca^2+^ + Mg^2+^) − (Cl^−^ + SO_4_^2−^ + H_2_PO_4_^−^) is not valid in adult cattle with marked ketonuria.^18^ The BHB concentration in urine could be as high as 12 mmol/L in severely ketotic animals^44^ as the urine ketone concentration is approximately 4 times that of the plasma.^45^ In the present study the highest measured BHB concentration in the plasma was 4.4 mmol/L in one case in the LDA group. The pH in the urine was therefore probably influenced by the ketonuria. Ketosis is the most accompanying disease in the LDA group, as it is a common disease in the transition period postpartum. In the exploratory forward stepwise multivariable regression analysis in the present study, venous blood BHB concentration emerged as a potential predictor of both venous blood pH and urinary pH, with higher BHB concentrations associated with lower pH values.

In addition to ketosis, the cows in the LDA group had other concomitant diseases such as metritis, endometritis, and mastitis. These diseases might also have contributed to changes in electrolyte concentrations in blood and urine, as they influence food intake, milk yield and the composition of mastitis milk differs from normal milk.^46^^,^^47^

Only 8 of the 20 cows with LDA had acidic urine (pH 5-6). Comparable results were reported in another study. With 1982 included animals 50% of cows with abomasal volvulus had acidic urine. In comparison, only 33% of the cows with LDA and 38% of the cows with right-sided abomasal displacement had a paradoxical aciduria.^30^

As pCO_2_ influences the pH of the urine as well, a loss of CO_2_ into the air could have led to an increase in urinary pH. A decrease of urinary *p*CO_2_ from 50 mm HG to 25 mm HG after sampling collection will increase urinary pH by approximately 0.06 pH units.^18^ The samples of the LDA group were analyzed directly, the samples of the control group were transported in a syringe with an airtight lid. Therefore, the loss of CO_2_ via the air was most likely very low and led to a very small increase in urinary pH. A major limitation of this study is the difference in DIM between the 2 groups (mean 12.7 vs. 204.4 days). As the cows with left displaced abomasum were patients of the University Veterinary Hospital Zurich, whereas the control animals originated from a small study herd, it was not possible to form comparable pairs at similar stages of lactation for group comparison. Consequently, differences observed in several electrolytes and other measured variables might partly be explained by the stage of lactation rather than by LDA itself. The following variables observed in the present study could at least partly be attributable to the transition period: higher plasma BHB, lower plasma calcium, phosphorus, sodium, and—at least in part—the reduced plasma potassium concentrations in cows with LDA compared with the control group.^32^^,^^37^^,^^39^^,^^44^ There are significant variations of electrolyte fractional excretion in the transition period from 2 weeks pre partum to 4 weeks postpartum in cows.^48^ In this study, all cows in the LDA group were in this period which might have influenced the results of the electrolyte concentrations in this group.

Sixty-one percent of the variance in urine pH could be explained by urinary potassium and sodium, venous BHB concentrations, and group affiliation. Sulfate (SO_4_) also has a significant influence on urine pH, as the urinary SID can be calculated as follows: SID = K^+^ + Na^+^ + Mg^2+^ + Ca^2+^—Cl^−^ − SO_4_^2−^.^18^ However, in the present study, urinary sulfate was not measured, and therefore no conclusions can be drawn regarding its contribution.

One limitation of this study is the measurement of the urinary pH with the urine stripe (Roche Combur Test) instead of a pH meter, as the pH meter would have shown more accurate results.^49^ In addition, the bicarbonate could have influenced the results of the chloride concentration in the urine using the indirect potentiometry. This could have led to incorrectly low chloride concentrations being measured with the cobas® 6000 analyzer series cobas® c 501 module (Roche Diagnostics, Basel, Switzerland) Urine chloride measurements were likely markedly overestimated in the presence of high urine bicarbonate concentrations, as observed in alkaline urine, and underestimated when urine bicarbonate concentrations were low, as seen in acidic urine.^50^^,^^51^ Another limitation of the study is the measurement of BHB using a handheld ketometer. The issue of linearity is a known concern with point-of-care ketone meters. Measurement using a laboratory reference method, such as BHB dehydrogenase-based assays, would have been more accurate and would have allowed for a more precise assessment of the influence of BHB concentration on pH.^52^

A limitation of the present study is the relatively small sample size (*n* = 40), which could have constrained the statistical power and stability of the multivariable regression models. Although commonly cited guidelines suggest a minimum of 10-15 observations per predictor variable, our models approached the lower bound of these recommendations, particularly for the urine pH analysis including 4 retained predictors. Consequently, the risk of model overfitting and coefficient instability cannot be excluded. While the observed partial *R*^2^ values suggest moderate effect sizes, these estimates should be interpreted with caution given the potential for sampling variability in small cohorts. Future studies with larger populations are warranted to confirm the robustness and reproducibility of these associations.

Summarized, electrolytes like chloride and sodium but also BHB in the venous blood and potassium in the urine have a great effect on the pH variability. These acid base disturbances can be explained with the physiochemical approach, although no significant differences in venous SID and SIG could be demonstrated in the present study.

Although a few mechanisms could be confirmed using the physicochemical approach, and relationships between electrolyte changes and pH variability could be described, further studies are needed to gain a deeper understanding of the paradoxical aciduria that sometimes occurs with LDA.

## Supplementary Material

sub_material_ready_aalag177

## Off-label antimicrobial declaration

Authors declare no off-label use of antimicrobials.

## Institutional animal care and use committee or other approval declaration

Approved by the veterinary authorities of the Kanton Zurich, Switzerland (approval number ZH 192/18).

## Abbreviations

A^−^: total net negative charge of blood proteins
AG: anion gap
A_tot_: total plasma concentration of nonvolatile weak acids
BE: base-excess
BHB: beta-hydroxybutyrate
BS: Brown Swiss
HCO_3_^−^: bicarbonate
HF: Holstein Friesian
LDA: left-sided abomasal displacement
mmol: millimoles
NH_4_^+^: ammonium
*p*CO_2_: partial pressure of carbon dioxide
*p*O_2_: partial pressure of oxygen
PO_4_: phosphorus
RH: Red Holstein
SI: Simmental
SID: strong ion difference
SIG: strong ion gap
TP: total protein
