# Supplementary material for: Association of electrolyte concentrations with pH of blood and urine of cows with left-sided abomasal displacement
Source: J Vet Intern Med. 2026 Aug 24;40(4):aalag177. doi: 10.1093/jvimsj/aalag177 (PMC13502405; doi:10.1093/jvimsj/aalag177)
Supplement: Supplementary file 1 [file sub_material_ready_aalag177.docx]

**Supplemental material**

**Table 1: Age and breed of the cows**

| **Number** | **Group** | **Age (years)** | **Breed** | **Accompanying diseases** |
| --- | --- | --- | --- | --- |
| **1** | Control | 4.2 | BS | - |
| **2** | Control | 6.3 | BS | - |
| **3** | Control | 3.1 | BS | - |
| **4** | Control | 5.2 | BS | - |
| **5** | Control | 6.5 | HO | - |
| **6** | Control | 4.7 | HO | - |
| **7** | Control | 4.0 | BS | - |
| **8** | Control | 4.1 | HO | - |
| **9** | Control | 3.9 | HO | - |
| **10** | Control | 7.5 | RH | - |
| **11** | Control | 3.6 | HO | - |
| **12** | Control | 9.0 | RH | - |
| **13** | Control | 2.8 | HO | - |
| **14** | Control | 2.7 | HO | - |
| **15** | Control | 3.0 | RH | - |
| **16** | Control | 2.7 | HO | - |
| **17** | Control | 6.1 | BS | - |
| **18** | Control | 3.7 | BS | - |
| **19** | Control | 4.7 | BS | - |
| **20** | Control | 3.8 | RH | - |
| **21** | LDA | 6.8 | BS | Metritis, paraphlebitis of the udder vein |
| **22** | LDA | 5.3 | Mix | Metritis, mange, ketosis |
| **23** | LDA | 8.8 | SI | Metritis, claw disease, ketosis |
| **24** | LDA | 2.3 | HO | Mastitis quarter C, ketosis |
| **25** | LDA | 5.0 | HO | Ketosis |
| **26** | LDA | 4.8 | RH | *Dicrocoelium dendriticum,*ketosis |
| **27** | LDA | 3.0 | HO | Metritis, ketosis |
| **28** | LDA | 4.6 | RH | Ketosis |
| **29** | LDA | 4.6 | SI | Endometritis, ketosis |
| **30** | LDA | 4.9 | HO | Metritis, ketosis |
| **31** | LDA | 3.8 | SI | Non |
| **32** | LDA | 2.7 | HO | Subclinical masitis quarter B and D, endoparasitosis, ketosis |
| **33** | LDA | 2.2 | HO | Ketosis |
| **34** | LDA | 4.9 | RH | Ketosis |
| **35** | LDA | 2.2 | HO | Milk stone quarter A, ketosis |
| **36** | LDA | 2.4 | RH | Metritis, ketosis |
| **37** | LDA | 5.1 | RH | Hepatolipidosis, ketosis |
| **38** | LDA | 7.4 | HO | Abomasal ulcer type 3 (post surgery), ketosis |
| **39** | LDA | 5.1 | HO | Ketosis |
| **40** | LDA | 5.5 | RH | Non |
